# Retinal alterations in a pre-clinical model of an autism spectrum disorder

**DOI:** 10.1186/s13229-019-0270-8

**Published:** 2019-04-15

**Authors:** Elisa Maria Guimarães-Souza, Christina Joselevitch, Luiz Roberto G. Britto, Silvana Chiavegatto

**Affiliations:** 1 Department of Physiology and Biophysics, Biomedical Sciences Institute, University of São Paulo, Av. Prof. Lineu Prestes, 1524, São Paulo, SP 05508-000 Brazil; 2 Department of Experimental Psychology, Psychology Institute, University of São Paulo, Av. Prof. Mello Moraes, 1721, São Paulo, SP 05508-030 Brazil; 3 Department of Pharmacology, Biomedical Sciences Institute, University of São Paulo, Av. Prof. Lineu Prestes, 1524, São Paulo, SP 05508-000 Brazil; 4 Department and Institute of Psychiatry, Clinics Hospital (HCFMUSP), University of São Paulo Medical School, Rua Dr. Ovidio Pires de Campos, 785, São Paulo, SP 05403-903 Brazil

**Keywords:** Autism, Vision, Retina, Glutamate, GABA, Valproate, Neurodevelopment, Adolescence

## Abstract

**Background:**

Autism spectrum disorders (ASD) affect around 1.5% of people worldwide. Symptoms start around age 2, when children fail to maintain eye contact and to develop speech and other forms of communication. Disturbances in glutamatergic and GABAergic signaling that lead to synaptic changes and alter the balance between excitation and inhibition in the developing brain are consistently found in ASD. One of the hallmarks of these disorders is hypersensitivity to sensory stimuli; however, little is known about its underlying causes. Since the retina is the part of the CNS that converts light into a neuronal signal, we set out to study how it is affected in adolescent mice prenatally exposed to valproic acid (VPA), a useful tool to study ASD endophenotypes.

**Methods:**

Pregnant female mice received VPA (600 mg/kg, *ip*) or saline at gestational day 11. Their male adolescent pups (P29–35) were behaviorally tested for anxiety and social interaction. Proteins known to be related with ASD were quantified and visualized in their retinas by immunoassays, and retinal function was assessed by full-field scotopic electroretinograms (ERGs).

**Results:**

Early adolescent mice prenatally exposed to VPA displayed impaired social interest and increased anxiety-like behaviors consistent with an ASD phenotype. The expression of GABA, GAD, synapsin-1, and FMRP proteins were reduced in their retinas, while mGluR5 was increased. The a-wave amplitudes of VPA-exposed were smaller than those of CTR animals, whereas the b-wave and oscillatory potentials were normal.

**Conclusions:**

This study establishes that adolescent male mice of the VPA-induced ASD model have alterations in retinal function and protein expression compatible with those found in several brain areas of other autism models. These results support the view that synaptic disturbances with excitatory/inhibitory imbalance early in life are associated with ASD and point to the retina as a window to understand their subjacent mechanisms.

**Electronic supplementary material:**

The online version of this article (10.1186/s13229-019-0270-8) contains supplementary material, which is available to authorized users.

## Background

Autism spectrum disorders (ASD) are a diverse group of developmental disorders, characterized by early-appearing social communication deficits and unusual sensory-motor behaviors (for a review, see [1]). In fact, symptoms are usually noticed in early childhood, at about age 2, when children fail to maintain eye contact and to develop speech and other forms of communication. They may also develop abnormal behavior that varies greatly and may determine autism severity, such as fixed interest in specific objects, repetitive movements, and cognitive impairment [1, 2].

There are several etiologies proposed for ASD. Some patients carrying mutations in a single gene, such as Phelan-McDermid (SHANK3), fragile X (FMRP), Rett (MeCP2), and tuberous sclerosis (TSC1/2) display autistic-like behaviors [3]. However, they are rare monogenic syndromes and represent only a small proportion of all ASD cases diagnosed. Most ASD occurrences are classified as multigenic/complex or idiopathic. Such patients may carry more than one genetic abnormality at the same time, translating into synaptic disturbances during neurodevelopment [3]. Accordingly, dysregulation in synaptic transmission and neural circuits due to the imbalance between excitation and inhibition (E/I) is hypothesized to contribute to ASD [4]. Disturbances in glutamatergic and/or GABAergic signaling are associated with ASD in a multitude of studies and may represent a convergence of small perturbations in multiple genes and proteins. For example, glutamic acid decarboxylase (GAD), the enzyme that produces GABA from glutamate, is found to be reduced in ASD patients due to an increased binding of the gene MeCP2 to its promoter [5]. It is therefore likely that E/I imbalances in ASD are caused by changes in the availability of glutamate and/or GABA.

In parallel with the genetic evidence of etiologically distinct conditions for ASD, environmental factors may also play a role in the increase prevalence of autism in the past decades. Increased exposure to pollution, pesticides, drugs, viral infections, and autoimmune diseases during pregnancy are associated with a higher risk of autism for embryos in utero [6]. Valproic acid (VPA), an affordable and stable drug used to treat epilepsy and bipolar disorder, may be one such environmental factor [7, 8]. VPA acts on both glutamatergic and GABAergic systems in order to decrease excitability: it antagonizes NMDA receptors, blocks sodium and calcium voltage-gated channels, and inhibits GABA transaminase, consequently increasing the concentration of GABA in the synaptic cleft. VPA is also a potent inhibitor of histone deacetylase (HDAC) and of glycogen synthase kinase-3 beta (GSK-3 beta) [9]. However, VPA is very teratogenic [10], and several women who inadvertently continued taking VPA during their pregnancy gave birth to children with cranial malformations, specific facial characteristics, cognitive problems, and/or autism [11]. These observations led researchers to consider VPA a good pharmacological tool to induce a phenotypic ASD model [9, 12, 13]. At the same time, this pharmacologically induced model has the advantage to represent what would be a syndrome of environmental etiology, allowing us to identify similarities and differences between this model and the more defined genetic syndromes [14].

In this work, we have investigated the retina of the VPA-induced model, since one of the hallmarks of ASD is hypersensitivity to sensory stimuli [15–18], and the retina is the primary sensory detector for vision. For example, ASD patients apparently perform better than usual in some visual tasks, and worse in others [19]. It has also been shown that certain types of visual stimulus, such as computer screen lights, capture the attention of individuals affected by ASD more than would be expected for neurotypical people [20]. Additionally, because the retina is part of the central nervous system (CNS), it uses mainly glutamate and GABA to transmit and modulate visual signals [21] and produces most, if not all, neurotransmitters found in the brain. It is therefore likely that the retina is also affected by the developmental disturbances that lead to autism. Our results show alterations in both protein expression and in the full-field scotopic electroretinogram (ERG) of adolescent mice (P30—for a definition of adolescence in rodents, see [22]) prenatally exposed to VPA, in addition to the same autistic-like behavioral features described elsewhere (for a review, see [13]).

## Methods

### Animal care

C57BL/6 mice were provided by the Animal Facility of the Department of Physiology and Biophysics (Biomedical Sciences Institute, University of São Paulo). Animals were kept in microisolated policarbonate cages (up to five per cage) in ventilated stands (Alesco, Brazil) located in a room with controlled temperature (± 22 °C) and a 12/12 h light cycle (lights on at 7 am), with water and feed *ad libitum*. All experiments were conducted between 7 am and 12 pm to avoid circadian differences that could induce additional variability in our results.

### VPA injection in pregnant mice

Adult female mice had their estrous cycle monitored and, when in proestrus, were housed with an age-matched male overnight. They were removed in the next morning, and their weight and vaginal smears were monitored; females were considered pregnant if they had gained about 3 g on day 7 after being with the male (E7) and were constantly in diestrus. On E11, 15 females were injected *ip* with either 100 μL of a 0.9% NaCl solution (control group, referred to as “CTR”) or 600 mg/kg of VPA sodium salt (P4543, Sigma Aldrich) diluted in 100 μL of a sterile 0.9% NaCl solution (experimental group, referred to as “VPA”). The final pH value of the VPA solution (6.05) was calculated from the pKa of VPA (4.6) and the concentration of the *ip* injection (36 μM of sodium valproate). This pH is within the acceptable range (4.5–8) for non-buffered solutions via all routes of administration in the mouse [23]. Therefore, pH adjustment of the VPA solution was not necessary. After birth, only male pups were used for our experiments, since female mice prenatally exposed to VPA appear to have their social interaction scores preserved in comparison to males [24, 25].

Twelve pregnant females (median 152 days old—from 65 to 165—at the injection day) yielded viable litters with 39 male pups that were weaned at P21. At P29, adolescent males were submitted to two behavioral experiments (the open field test and the social interaction test), and between P30 and P35, some of these mice were subjected to full-field scotopic electroretinograms (ERGs) to assess their retinal function, while some of them were used for immunoblotting and immunohistochemistry. Some mice were exclusively used for immunoassays. Detailed information regarding the number of animals in each group and type of experiment is given in Additional file 1: Figure S1.

### Open field test

The open field consisted of a wooden arena covered with white Formica measuring 66 × 66 × 30 cm, and its floor was divided into 6 × 6 cm squares. Each mouse was placed in the center of the arena and allowed to explore the environment freely for 5 min. Experiments were recorded with a Nikon P3200 camera. Videotrack software (ViewPoint, France) was used to analyze the experiments; evaluated behaviors were (i) total locomotion, (ii) locomotion in the central area, and (iii) time in the central area. See Additional file 1: Figure S2 for details about the delimitation area.

### Social interaction test

The social interaction test we used was a modified version of the three-chamber social interaction test. We adapted this assay for adolescent mice in our lab using only two contiguous and connected chambers (40.5 × 20 × 22 cm height each) of the sociability cage (Noldus, Wageningen, The Netherlands). The salient sensory cues that contribute to social approach performance are primarily olfactory in nature, with visual cues appearing to play a remarkably minor role [26]. The subject mouse was placed in an empty and clean chamber and was free to explore the whole apparatus for a 3-min habituation period. It was then removed, while a perforated acrylic cylinder containing a naïve male adolescent mouse of approximately the same size was introduced in the other chamber. The subject mouse was subsequently placed back in the empty chamber and allowed to explore for another 3 min. We evaluated the subject’s latency to enter the interaction area, the time during which it stayed in the interaction area, and the locomotion around the cylinder with the novel animal (see Additional file 1: Figure S2 for area delimitation). The frequency of sniffing between the cylinder bars towards the novel mouse was considered social exploration. Sessions were recorded and analyzed by Videotrack software (ViewPoint), and the nose pokes were manually quantified from the videos in a blind fashion.

### Full-field scotopic electroretinogram

In order to evaluate the function of outer retinal neurons such as photoreceptors (rods and cones) and depolarizing bipolar cells (ON BCs), as well as amacrine cells (ACs) in the inner retina, full-field (*Ganzfeld*) scotopic electroretinograms (ERGs) were measured in animals between P30 and P35 (details in Additional file 1: Figure S1). Mice examined with the ERG were not used for immunoassays or immunohistochemistry, since the anesthetic drug used for the ERG interferes with NMDA receptors [27], which are abundantly present in the inner retina [28].

On the day before the experiment, animals were weighted and left in the experiment room to dark adapt overnight (~ 16 h), with feed and water *ad libitum*. On the day of the ERG, the mice were anesthetized under dim red light (*λ* = 680 nm, 6.44 cd/m^2^) with an intraperitoneal injection of 80 mg/kg ketamine and 2.5 mg/kg xylazine diluted in 150 μL of a sterile 0.9% NaCl solution and laid on a heated table (~ 39 °C) to maintain their body temperature. All subsequent procedures were performed with lights off and using infrared goggles (Night Cougar, ATN).

We used eye drops to locally anesthetize (0.5% proxymetacaine hydrochloride, Anestalcon, Alcon) and dilate (0.5% tropicamide, Mydriacyl, Alcon) the animals’ pupils. Five electrodes (Roland Consult, Germany) were connected to their body: one needle electrode at the base of the tail as ground, two needle electrodes at each side of the forehead as references, and two gold ring electrodes (0.25 mm wire; 2.5 mm diameter) on the corneas for recording. A *Ganzfeld* LED stimulator (Q450 SC Roland Consult, Germany) was used to produce homogeneous light stimuli over the entire retina (*λ*_max_ = 459 nm), ranging from − 2.01 to 4.09 log photons*μm^−2^*s^−1^ in ~ 0.5 log increments. Experiments were controlled, amplified, and digitized by a computerized system (RetiPort, Roland Consult, Germany). For each light intensity, the mean response to at least five presentations was calculated. ERG sessions typically lasted 40–60 min; data was discarded whenever (i) electrode contact was deemed inadequate, (ii) the anesthesia wore out during the experiment, or (iii) the animal came to pass during the experiment. In the first case, only the data from the affected eye was discarded. In the latter cases, all records of that animal were discarded.

**Fig. 1:**
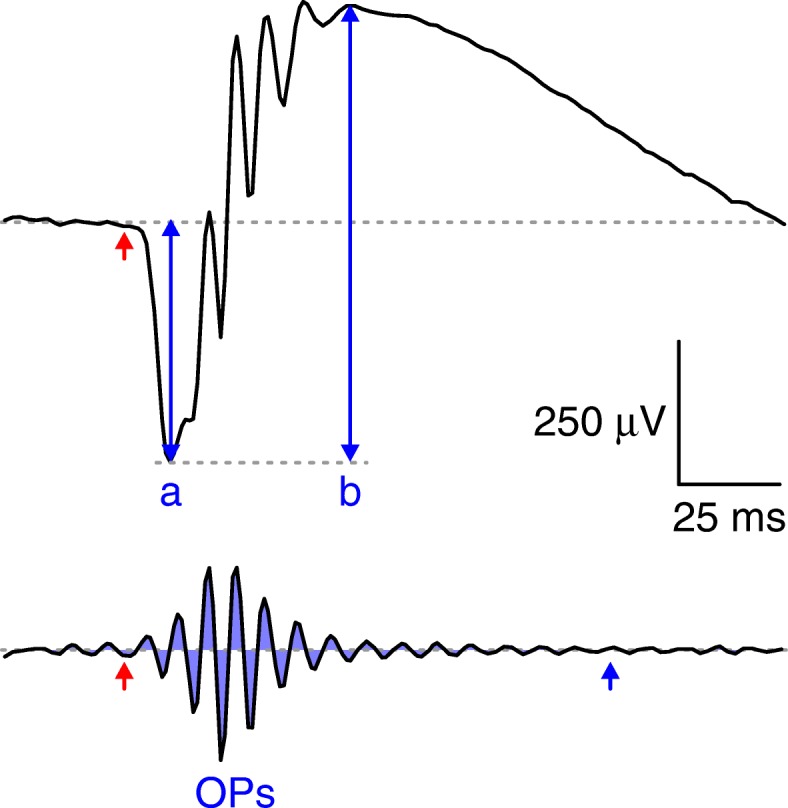
ERG analysis. Representative ERG trace showing the different retinal waves in response to a full-field light stimulus (timing indicated by the red arrow). A-wave and b-wave amplitudes are represented by blue arrows, and the total OP area is the absolute sum of the areas shaded in blue between 30 ms (stimulus onset) and 150 ms (end of oscillations, indicated by the blue arrow)

Data were further processed using macros programmed in OriginPro 8 software (OriginLab). A notch digital filter at 60 Hz was used to eliminate noise originating from the electrical network. A-wave amplitudes, which reflect photoreceptor (rod and cone) activity, were measured directly on the raw traces from the baseline (dotted gray line in Fig. 1, upper panel) until the trough of the trace. B-wave amplitudes, which are generated by ON BCs and provide a useful estimate of signal transmission between photoreceptors and second-order neurons, were subsequently measured from the trough of the a-wave to the peak of the trace (Fig. 1, upper panel) after treatment with a 60-Hz low-pass filter to eliminate oscillatory potentials (OPs). Finally, OPs, generated by the joint activity of different AC types and providing a readout of signal transmission from bipolar cells to the inner retina, were quantified by calculating the absolute areas of the traces (i.e., the sum of absolute trapezoid values, in μV*ms) from 30 ms (stimulus onset, red arrow in Fig. 1, lower panel) to 150 ms (end of oscillations) after isolation with a 120-Hz high-pass filter.

Amplitudes of the a- and b-waves were fitted by a sigmoid function of the form:

$$ V={V}_{\mathrm{max}}\frac{I^n}{k^n+{I}^n}, $$

in which *V*_max_ is the maximal response amplitude, *k* is the intensity required to evoke 50% of *V*_max_, *n* is the slope, and *I* is the stimulus intensity. This relation is called *Hill function* and is a variation of the *Michaelis-Menten* equation originally developed for enzyme kinetics [29]. Since the area-intensity relationship of OPs has a biphasic nature, two light intensities were compared: (i) an intermediate intensity (intensity #8, 1.56 log photons*μm^−2^*s^−1^), corresponding to a first plateau in this relationship and well within the scotopic (rod-driven) range for the mouse [30] and (ii) the last intensity (intensity #13, 4.09 log photons*μm^−2^*s^−1^), which is in the mesopic/photopic range and reflects therefore cone-driven activity [30].

### Immunoblotting

P30–35 mice were euthanized with an overdose of isoflurane and their eyecups were removed. Retinas were dissected, collected in RIPA buffer and sonicated. Samples were centrifuged at 12000×*g* and the supernatant was collected. We used the Bradford method [31] to measure protein content in the samples, and then added alkaline denaturing buffer. Samples were heated at 60 °C for 10 min, and 60 μg of each protein sample were loaded into 8% polyacrylamide gels for electrophoresis. Proteins were transferred to nitrocellulose membranes, incubated with primary antibodies (Table 1, dilutions referenced as WB) overnight, rinsed, and incubated with HRP-conjugated secondary antibodies. We used a chemiluminescence kit (Clarity ECL, Biorad) to reveal the bands, and the membrane was imaged with Image Studio Digits 4.0 software together with the c-Digit scanner (both from LI-COR Biosciences) to measure the optical density (OD) of each band. It uses an algorithm that subtracts the background from the chemiluminescent signal captured by the device. Measurements are therefore reliable, because they are independent of image contrast, brightness, or exposure. Membranes were stripped with a glycine solution (pH 2.2) for reprobing. Lastly, all membranes were incubated with beta-actin antibody (A5316, Sigma), and beta-actin bands were used for normalization.

**Table 1:** Information about primary antibodies used in this study

Antibody	Catalog #/company	Species	Dilution
Anti-beta-actin	A5316	Mouse	1:50000 (WB)
Anti-synapsin-1 (SYN-1)	AB1543/Millipore	Rabbit	1:500 (IHC); 1:5000 (WB)
Anti-mGluR5	ab76316/Abcam	Rabbit	1:200 (IHC); 1:1000 (WB)
Anti-FMRP	ab17722/Abcam	Rabbit	1:200 (IHC); 1:1000 (WB)
Anti-GABA	A2052/Sigma	Rabbit	1:1000 (IHC)
Anti-GAD	AB1511/Millipore	Rabbit	1:200 (IHC); 1:1000 (WB)
Anti-GAT-1	ab426/Abcam	Rabbit	1:500 (IHC)

*IHC* immunohistochemistry, *WB* Western blot

### Immunohistochemistry

Eyecups were fixed in 4% paraformaldehyde diluted in 0.1 M phosphate buffer (pH = 7.2, PB). Tissues were cryoprotected in a sucrose gradient (10%, 20%, and 30%), sectioned in a cryostat at 12 μm and placed onto gelatinized glass slides. CTR and VPA sections were collected on the same slide to ensure the same immunohistochemical reaction for both samples. Sections were washed with PB and then blocked with 5% normal donkey serum for 1 h. After three washes with PB, sections were incubated with the primary antibodies listed in Table 1 (dilutions referenced as IHC) overnight. The next day, sections were washed three times and incubated with a fluorescent secondary anti-rabbit antibody raised in donkey (Alexa 594, Invitrogen) for 2 h. Sections were then washed three times and mounted with Vectashield (VectorLabs) containing DAPI.

Images were captured with a DXM 1200 camera coupled to an Eclipse E1000 fluorescence microscope using a × 40 objective (all from Nikon, Japan). CTR and VPA photographs were taken on the same day using the same resolution and exposure settings to prevent artifacts induced by time-dependent fluorescence decay. Analysis was performed using the RGB value plugin of ImageJ software (NIH). We used the polygon tool to draw a selection around each retinal layer (outer plexiform layer, OPL; inner nuclear layer, INL; inner plexiform layer, IPL; ganglion cell layer, GCL) and measured the red value. We analyzed ten images from each retina, totalizing at least 50 images for each group. Individual values obtained from single images were summed to account for the whole retina. It should be noted that the final number of eyes used for IHC is smaller than the one for immunoblotting because we only analyzed images from eyes that yielded at least ten perfectly labeled images.

### Statistical analyses

Statistical tests were performed on the ERG data using Origin Pro 8 software (OriginLab) and Excel (Microsoft). Data normality was checked with the Shapiro-Wilk test. For non-normal data, two-tailed Mann-Whitney tests were used to compare results; in these cases, medians are reported instead of means. For normal data, the *F* test was used to analyze the equality of variances, followed by the appropriate unpaired two-tailed *t* test; in these cases, the means ± SDs are reported. For all statistic tests, a confidence interval of 0.95 was initially chosen (i.e., *α* = 0.05). Whenever more than one comparison was performed with the same dataset, Bonferroni correction was applied according to the number of hypotheses tested, by adjusting either the level of significance *p* or *α*. For non-normal data, *p* was divided by the number of comparisons (i.e., for two comparisons using the same dataset, the significance level was reduced from *p* = 0.05 to *p* = 0.05/2 = 0.025). For normal data, *α* was divided by the number of comparisons, and *p* remained 0.05.

For the remainder of the data, after checking for data normality with the Shapiro-Wilk test, two-tailed unpaired *t* tests were performed with Prism 6 software (Graphpad); results were considered significantly different when *p <* 0.05. Immunohistochemistry analyses were done with two-tailed paired *t* tests (*p <* 0.05), to compare results obtained from CTR and VPA in the same slide and immunohistochemical procedure.

## Results

### Mice from VPA-injected dams display different physical characteristics from control mice

Our first approach was to physically inspect all pups. We observed features in the VPA prenatally exposed mice that were similar to those already described in the literature: some pups showed hair loss; some exhibited crooked tails, which probably indicates problems in the development of the CNS [32, 33]; and some pups were smaller and weighed less than the average for control animals (Additional file 1: Figure S3). These characteristics were either found in the whole litter or were restricted to one or two pups in the litter. All males were used for the following experiments, independent of size, weight, or appearance.

### Behavioral evaluation of the VPA-exposed adolescent mice confirms anxiety and anti-social features

**Fig. 2:**
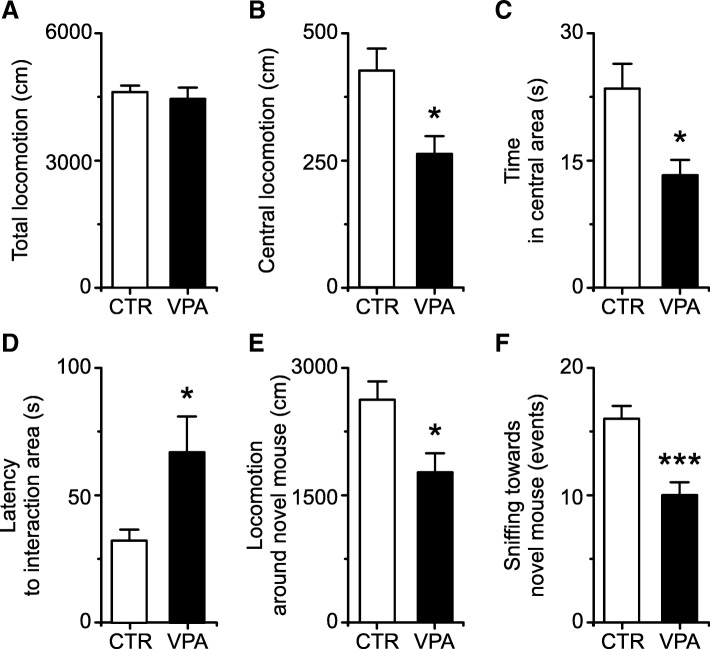
Behavioral phenotype of adolescent (P29) VPA male mice. **a**–**c** In the open field test, VPA mice (*n =* 9; black bars) show similar total locomotor activity and higher anxiety-like behaviors than CTR (*n =* 13; white bars); **d**–**f** in the social interaction test, VPA mice (*n =* 11; black bars) show reduced interest to approach and to investigate (sniffing events) the novel animal than CTR mice (*n =* 14; white bars). Mean ± SEM, *p < 0.05, and ***p < 0.001. In Additional file 1, see Figure S2 for area delimitation and representative videos for the open field (Videos S1 and S2) and social interaction test (Videos S3 and S4)

VPA mice did not show ambulatory deficits in the open field test [total distance traveled: 4612 ± 562 cm for CTR (*n =* 13; one animal was not filmed due to technical problems) *vs.* 4449 ± 800 cm for VPA (*n =* 9; two animals were not filmed due to technical problems); mean ± SD; *p =* 0.580; Fig. 2a]. However, in the central area, VPA animals ambulated less (426 ± 156 cm for CTR *vs.* 262 ± 106 cm for VPA; *p =* 0.012; Fig. 2b) and spent less time than CTR mice (23 ± 11 s for CTR *vs.* 13 ± 6 s for VPA; *p =* 0.015; Fig. 2c). The shorter time of VPA mice in the most aversive area of the open field was not due to different traveling speed (19 ± 4 cm/s for CTR *vs.* 20 ± 5 cm/s for VPA; *p =* 0.455). Conversely, animals of the VPA group preferred to explore the peripheral area for a longer time (229 ± 16 s for CTR *vs.* 245 ± 19 s for VPA; *p =* 0.046), what may be interpreted as a higher anxiety trait in these mice (Additional file 1: Videos S1 and S2).

In the social interaction experiment, VPA mice did not show specific interest for either chamber during the initial habituation period (before adding the novel animal), since all of them visited both chambers equally. Once the novel animal was introduced, VPA mice (*n* = 11) took a longer time in comparison to CTR mice (*n* = 14) to visit the chamber where the novel animal was placed (32 ± 16 s for CTR *vs*. 67 ± 47 s for VPA; *p =* 0.016; Fig. 2d). After the first interaction episode, VPA mice spent the same time as CTR mice in the interaction area (118 ± 24 s for CTR *vs*. 97 ± 32 s for VPA; *p =* 0.076). However, they ambulated around the novel animal significantly less than CTR animals, indicating a lower level of energy or interest to explore the cylinder with the novel mouse (2624 ± 803 cm for CTR *vs.* 1769 ± 747 cm for VPA; *p =* 0.012; Fig. 2e). We found a significant decrease in the number of times VPA animals approached the cage to sniff between bars towards the novel animal (social investigation) in relation to CTR (nose poke events, 16 ± 3 for CTR *vs.* 10 ± 4 for VPA; *p <* 0.001; Fig. 2f). There was no statistically significant difference between VPA and CTR in terms of total locomotion (5067 ± 1232 cm for CTR *vs.* 4210 ± 1342 cm for VPA; *p =* 0.110). In summary, these results suggest that VPA adolescents have reduced interest to explore and socialize with other mice, confirming that they phenotypically resemble individuals with ASD (Additional file 1: Video S3 and S4).

### ERGs of VPA-exposed animals are abnormal

As mentioned previously, VPA animals were significantly smaller and lighter than CTR mice (Additional file 1: Figure S3). Even though the dose of anesthetic was carefully calculated for each animal, the mortality of VPA mice during the ERG experiments was much higher than that of CTR (5 VPA animals *vs.* 0 CTR). Since the records of deceased animals were excluded from the analysis in order to prevent artifacts (see the “Methods” section), the final number of animals reported henceforth for the VPA group (*n* = 6 animals, 12 eyes) is smaller than that of the CTR group (*n* = 9 animals, 15 eyes; some records were excluded due to poor electrode contact, see the “Methods” section).

**Fig. 3:**
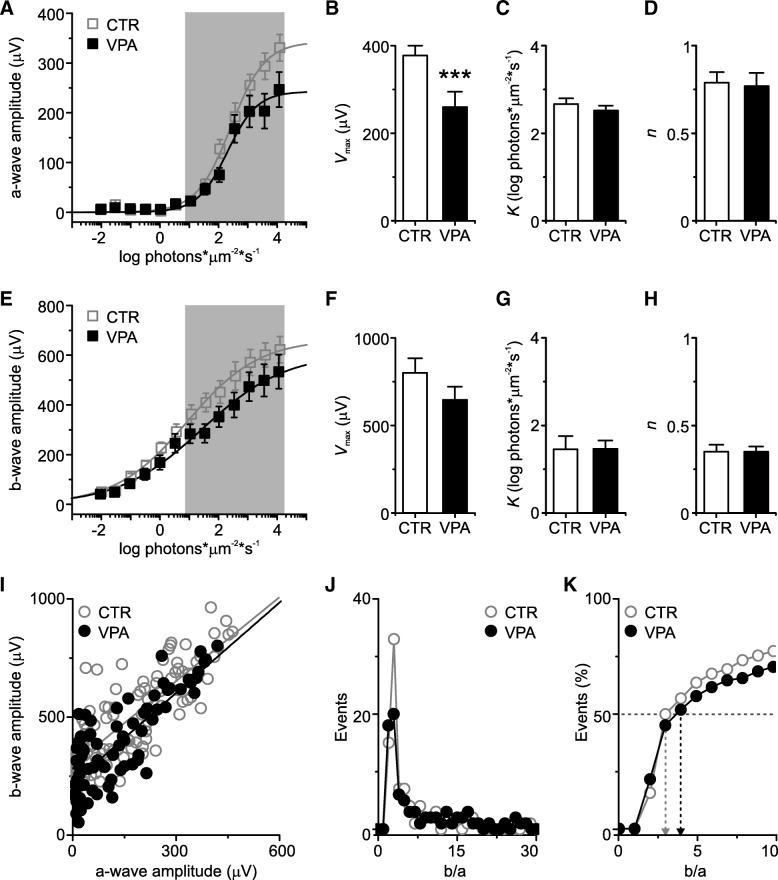
VPA animals have significantly smaller a-waves than CTR. **a** Semi-log plot of a-wave mean amplitudes obtained at each light intensity for CTR (open symbols, *n* = 8 animals, 15 eyes) and VPA animals (solid symbols, *n =* 6 animals, 12 eyes). The solid lines are *Hill* fits to the data points. The a-wave amplitudes of the VPA group are smaller, especially at higher stimulus intensities. **b** The mean maximal a-wave amplitude (*V*_max_) of VPA is smaller than that of CTR mice (***p < 0.001). **c**, **d** The mean semi-saturation constant (*k*) and slope (*n*) of the a-wave intensity-response relationship for CTR *vs.* VPA are not significantly different. **e** Semi-log plot of b-wave mean amplitudes obtained at each light intensity for CTR (open symbols, same *n* as in (**a**–**d**)) and VPA animals (solid symbols, same *n* as in (**a**–**d)**). The solid lines are *Hill* fits to the data points. Although b-waves are slightly smaller in the VPA group, the difference did not reach statistical significance. **f**–**h** The mean maximal b-wave amplitude (*V*_max_), semi-saturation constant (*k*), and slope (*n*) of the b-wave intensity-response relationship for CTR *vs.* VPA are not significantly different. For all panels, error bars are SEM. **i** Amplitudes of the b-wave *vs.* the corresponding a-wave amplitude in the same trace for CTR (open symbols) and VPA mice (solid symbols). Solid lines are linear fits to the data (CTR: adjusted *R*^*2*^ = 0.64; VPA: adjusted *R*^*2*^ = 0.66). **j** The distributions of the ratios between the b-wave and a-wave in the same trace for CTR (open symbols) and VPA mice (solid symbols) are very similar. **k** Cumulative distribution of the ratios between the b-wave and a-wave in the same trace for CTR (open symbols) and VPA mice (solid symbols). Dashed arrows indicate the median of each group (same color code as the respective symbols)

Although the ERG traces of VPA mice appeared at first glance similar to those of CTR animals, close inspection revealed significant differences between groups. Figure 3a presents the mean intensity-response relationship of the a-wave for CTR (open symbols) and VPA (solid symbols) mice and shows that a-wave amplitudes were smaller in the VPA group, especially at higher stimulus intensities (error bars are SEM, solid lines are *Hill* fits to the data points). Indeed, the mean a-wave *V*_max_ was significantly smaller for VPA animals (*V*_max,_ 377.3 ± 87.5 μV for CTR *vs*. 259.7 ± 121.1 μV for VPA; *p =* 0.007, two-tailed *t* test; Fig. 3b), indicating that photoreceptor activity is impaired in this group. On the other hand, the semi-saturation constant (*k*, 2.67 ± 0.50 log for CTR *vs.* 2.52 ± 0.39 log for VPA; *p =* 0.415, two-tailed *t* test; Fig. 3c) and slope (*n*, 0.79 ± 0.24 for CTR *vs.* 0.77 ± 0.26 for VPA; *p =* 0.839, two-tailed *t* test; Fig. 3d) of the a-wave intensity-response relationship were not significantly different between groups. Together, these results suggest that VPA exposure did not affect photoreceptor sensitivity (quantified by *k*) or the degree of cooperativity between stimulus increment and the rate of closure of ionic channels in the photoreceptor membrane (quantified by *n*), but may have affected other aspects of the photoreceptor intracellular machinery, such as the amplification or the inactivation of the phototransduction cascade (quantified by *V*_max_). Furthermore, since the a-wave receives mixed contribution from rods and cones [30, 34], which are active at low and high light levels, respectively, and the effect reported here is more pronounced at higher intensities, it seems likely that cones are more affected than rods in the VPA model.

The mean b-wave amplitudes of VPA mice (Fig. 3e, solid symbols) were slightly smaller at higher intensities than those of the CTR group (Fig. 3e, open symbols), albeit not significantly (error bars are SEM, solid lines are *Hill* fits to the data points). This would suggest that synaptic transmission between photoreceptors and ON BCs, as well as ON BC activity itself, were not only intact, but slightly supranormal, since they partially compensated for the smaller photoreceptor input: median *V*_max_ was 763.9 μV for CTR *vs*. 646.8 μV for VPA (two-tailed Mann-Whitney *U* = 114, *n*_1_ > *n*_2_; *p =* 0.256; Fig. 3e); *k* was 1.46 ± 1.15 for CTR *vs.* 1.47 ± 0.67 for VPA (*p =* 0.971, two-tailed *t* test; Fig. 3g); and median *n* was 0.31 for CTR *vs.* 0.34 for VPA (two-tailed Mann-Whitney *U* = 70, *n*_1_ > *n*_2_; *p =* 0.347; Fig. 3h). Despite the smaller a-waves and lack of significant difference between the b-wave amplitude of CTR and VPA animals, the overall relationship between the b-wave amplitude and a-wave amplitude was not significantly changed in the VPA model (Fig. 3i-k; the median b/a ratio of each group, indicated by the dashed arrows in the cumulative histogram of Fig. 3k, was CTR 2.99 *vs.* VPA 3.56, two-tailed Mann-Whitney *U* = 3771, *n*_1_ > *n*_2_; *p =* 0.62), indicating that BCs indeed do not compensate completely for the smaller photoreceptor input.

**Fig. 4:**
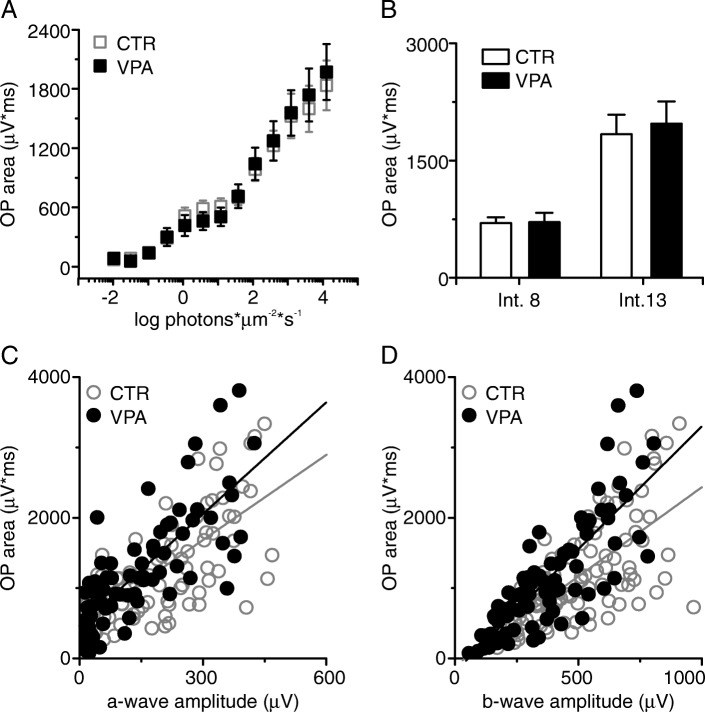
OP areas are similar in both groups. **a** Semi-log plot of mean OP areas (± SEM) obtained at each light intensity for CTR (open symbols, *n* = 8 animals, 15 eyes) and VPA animals (solid symbols, *n =* 6 animals, 12 eyes). Areas are similar at all intensities. **b** Mean OP areas obtained from CTR and VPA animals at light intensity 8 (1.56 log photons*μm^−2^*s^−1^) and at light intensity 13 (4.09 log photons*μm^−2^*s^−1^). There was no significant difference between groups. Error bars are SEM. **c** OP area *vs.* a-wave amplitude for CTR (open symbols) and VPA mice (solid symbols). **d** OP area *vs.* b-wave amplitude for CTR (open symbols) and VPA mice (solid symbols). OP areas in VPA mice are significantly larger in relation to the a-wave amplitudes (*p = 0.023) and b-wave amplitudes (***p = 0.0007) in the same trace than those of CTR animals

Finally, the mean OP areas in VPA mice (Fig. 4a, solid symbols) were similar to those in the CTR group (Fig. 4a, open symbols) throughout the whole range of light intensities tested (error bars are SEM), suggesting that the inner retina compensated further for the smaller photoreceptor input in VPA animals, since OPs result from the activity of ACs, which are neurons downstream of the BCs that generate the b-wave. At intensity #8, mean OP areas were 698.7 ± 298.5 μV*ms *vs.* 712.3 ± 356.3 μV*ms for CTR and VPA groups, respectively (*p =* 0.921, two-tailed *t* test, Fig. 4b); at intensity #13, mean OP areas were 1836.7 ± 874.4 μV*ms for CTR *vs.* 1971.6 ± 896.3 μV*ms for VPA (*p =* 0.725, two-tailed *t* test, Fig. 4b). Because the a-wave of VPA animals was subnormal (Fig. 3a, b), and the b-wave marginally subnormal (Fig. 3e, f), but the OP area was similar to that of CTR (Fig. 4a), the relationship between OP area and the amplitudes of the a- and b-waves was significantly altered in the VPA group. In Fig. 4c, OP area is plotted *vs.* a-wave amplitude and *vs.* b-wave amplitude (Fig. 4d) for CTR animals (open symbols) and VPA mice (solid symbols) and shows that in VPA-exposed animals OP areas are significantly larger than in the CTR group in relation to their corresponding a-wave amplitudes (medians = 7.70 for CTR *vs.* 9.71 for VPA, two-tailed Mann-Whitney *U* = 3123, *n*_1_ > *n*_2_; *p =* 0.023) and b-wave amplitudes (medians = 2.16 for CTR *vs.* 3.04 for VPA, two-tailed Mann-Whitney *U* = 2786, *n*_1_ > *n*_2_; *p =* 0.0007) in the same trace (= same animal, same eye, same intensity). Solid lines are linear fits to the data (left graph: CTR adjusted *R*^2^ = 0.53, VPA adjusted *R*^2^ = 0.62; right graph: CTR adjusted *R*^2^ = 0.46, VPA adjusted *R*^2^ = 0.66).

The latencies of the a- and b- waves and the kinetics of OPs were unaltered in VPA mice (data not shown). Taken together, these results suggest that photoreceptor function is significantly altered in VPA male mice at this stage in life and that there are adaptive mechanisms at play in both the outer and inner retina to compensate for their smaller photoreceptor responses, but with no effects on response kinetics.

### Decreased synapsin-1 expression in the retinas of prenatally VPA-exposed mice

**Fig. 5:**
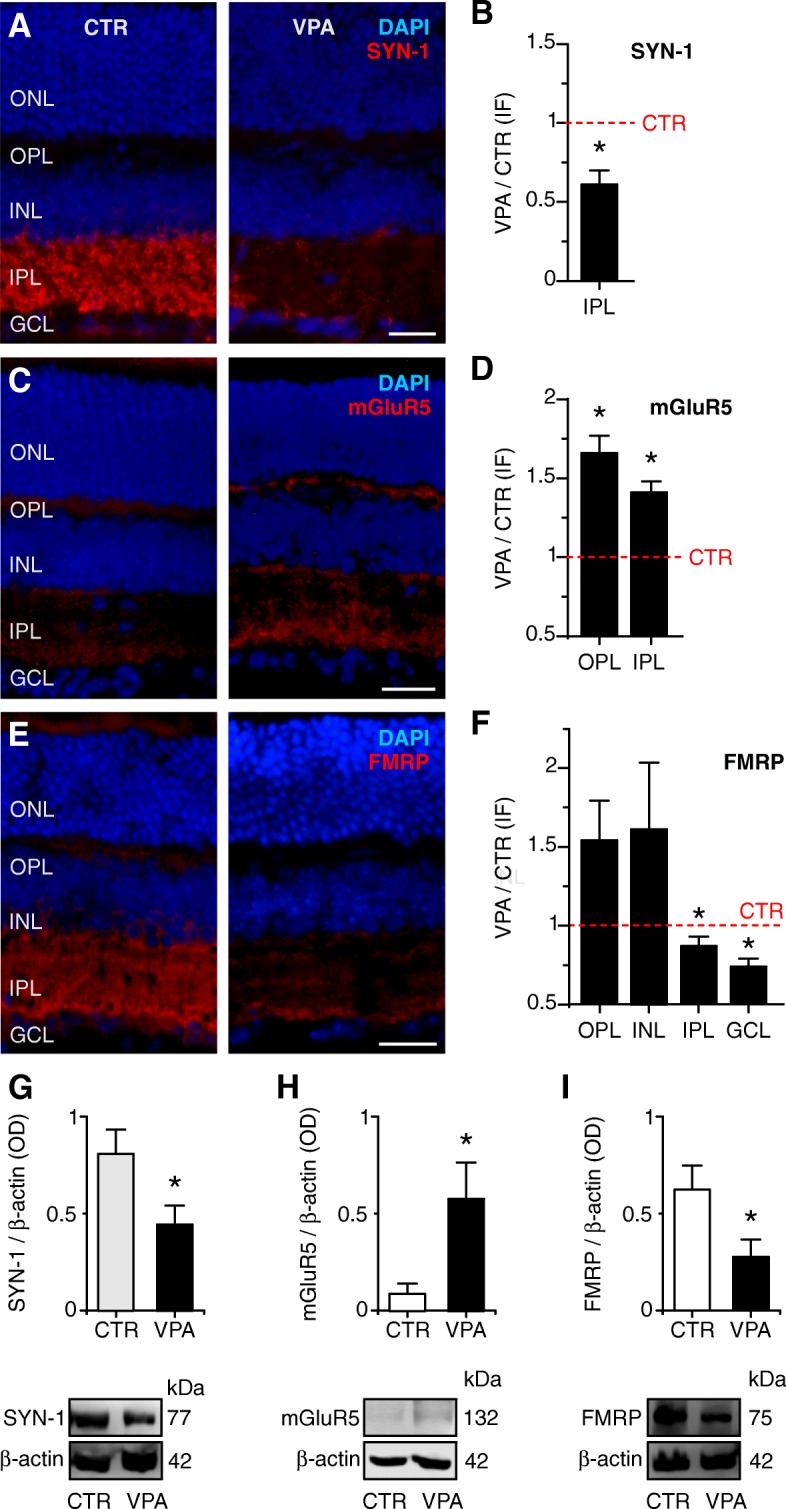
Altered expression of SYN-1, mGluR5, and FMRP in the VPA retina. **a** Photomicrographs of CTR and VPA retinal sections immunolabeled for synapsin-1 (SYN-1, red) and nuclei (DAPI, blue). SYN-1 immunoreactivity is located exclusively in the IPL in CTR and VPA mice and appears brighter in CTR. Scale bar = 25 μm. **b** The mean SYN-1 immunofluorescence signal of VPA retinal sections (normalized to CTR ± SEM; *n =* 5 each) is significantly smaller (*p = 0.021). **c** Photomicrographs of CTR and VPA retinal sections immunolabeled for mGluR5 (red) and nuclei (DAPI, blue). mGluR5 immunoreactivity is confined to both synaptic layers in CTR and VPA mice and appears brighter in VPA retinas. Scale bar = 25 μm. **d** The mean mGluR5 immunofluorescence of VPA retinal sections (normalized to CTR ± SEM; *n =* 6 each) is significantly larger in the OPL (*p = 0.010) and IPL (*p = 0.018). **e** Photomicrographs of CTR and VPA retinal sections immunolabeled for FMRP (red) and nuclei (DAPI, blue). FMRP immunoreactivity is strong in the IPL and GCL in CTR mice and fainter in VPA animals. Scale bar = 25 μm. **f** The mean FMRP signal in VPA retinal sections (normalized to CTR ± SEM; *n =* 5 each) is significantly reduced in the IPL (*p = 0.050) and GCL (*p = 0.020). The red dashed lines in panels **b**, **d**, and **f**) represent the value for CTR (= 1). **g** Top: Mean optical density (± SEM) of SYN-1 in immunoblots of CTR (white bar, *n =* 7) and VPA (black bar, *n =* 7) retinas, normalized to the beta-actin signal (*p = 0.026). Bottom: Representative SYN-1 bands (77 kDa) and the corresponding beta-actin bands (42 kDa) of CTR *vs.* VPA. **h** Top: Mean optical density (± SEM) of the mGluR5 signal in immunoblots of CTR (white bar, *n =* 7) and VPA (black bar, *n =* 6) retinas, normalized to the beta-actin signal (*p = 0.022). Bottom: Representative mGluR5 bands (132 kDa) and the corresponding beta-actin bands (42 kDa) of CTR *vs.* VPA. **i** Top: Mean optical density (± SEM) of the FMRP immunofluorescence in blots of CTR (white bar, *n =* 8) and VPA (black bar, *n =* 7) retinas, normalized to the beta-actin signal (*p = 0.050). Bottom: Representative FMRP bands (75 kDa) and the corresponding beta-actin bands (42 kDa) of CTR *vs.* VPA

We next evaluated the retinal expression of proteins related to the synaptic function that have been previously related to ASD. Since autism is considered a synaptic disease [35], the first molecule we analyzed was synapsin, a regulator of neurotransmitter release of conventional synapses. Synapsin is only expressed in the IPL, being absent in the OPL and in ribbon synapses in general [36]. As expected, the synapsin-1 (SYN-1) antibody labeled exclusively the IPL in both groups of animals (Fig. 5a). Labeling was diffuse and relatively homogeneous throughout the IPL in CTR and absent from the other retinal layers. VPA mice exhibited a decreased and less diffuse labeling pattern in the IPL when compared to CTR, with areas of concentrated fluorescence in the vitread portion of the IPL. Figure 5b quantifies this decrease in mean SYN-1 immunofluorescence in the VPA group after normalizing the signal from each VPA retinal section to its CTR pair in the same microscope slide and shows that fluorescence signal for the VPA group was 0.61 ± 0.21 times that of CTR retinas (CTR 577,648 ± 295,649 fluorescence units; VPA = 392,957 ± 294,104 units, *n =* 5 each, paired *t* test, *p =* 0.021, data in Additional file 1: Table S1). Consistent with this observation, immunoblots of VPA retinas also presented decreased SYN-1 content in comparison to CTR (0.91 ± 0.38 SYN-1/beta-actin OD for CTR, *n* = 7 *vs.* 0.50 ± 0.3 SYN-1/beta-actin OD for VPA, *n* = 7; *p =* 0.026), as shown in Fig. 5g.

### Retinas of VPA-exposed animals have increased mGluR5 expression

MGluR5 is a group I metabotropic glutamate receptor implicated in the pathogeny of ASD [37]. In both groups of animals, mGluR5 labeling was relatively faint and diffuse and confined to the synaptic layers of the retina (Fig. 5c). However, retinas from VPA mice presented higher mGluR5 immunoreactivity in both OPL (CTR 289,483 ± 107,387 fluorescence units *vs.* VPA 491,492 ± 222,137 units; *n* = 6 each; paired *t* test, *p =* 0.010) and IPL (CTR 357,344 ± 129,013 fluorescence units *vs.* VPA 514,333 ± 234,008 units; *n =* 6 each; paired *t* test, *p =* 0.018; individual data in Additional file 1: Table S2). In the IPL, labeling in VPA retinas seemed to be more concentrated at the inner IPL sublaminae. Figure 5d shows that the mean fluorescence signal of VPA retinas after normalization to the respective CTR pair in the same microscope slide was 1.66 ± 0.28 times that of CTR retinas in the OPL and 1.41 ± 0.18 higher than that of CTR in the IPL. Accordingly, immunoblots also presented increased mGluR5 content in VPA retinas in relation to CTR (CTR 0.10 ± 0.15 mGluR5/beta-actin OD, *n* = 7 *vs.* VPA 0.65 ± 0.52 mGluR5/beta-actin OD, *n* = 6; *p =* 0.022; Fig. 5h).

### Reduced retinal FMRP expression in the VPA model

Since mGluR5 regulates the local translation of FMRP (fragile X mental retardation protein), and *Fmr1*^*−/−*^ is a widely used animal model with ASD-like behaviors, we evaluated FMRP in our VPA mice [37]. In CTR retinas, FMRP signal was faint in the OPL and INL, and strong in the IPL and GCL (Fig. 5e); VPA mice exhibited a slightly (albeit not significantly) higher FMRP immunoreactivity in the OPL (CTR 182,911 ± 36,194 fluorescence units *vs.* VPA 272,634 ± 91,881 units; *n* = 5 each; paired *t* test, *p =* 0.115) and INL (CTR 132,693 ± 55,511 units *vs.* VPA 197,037 ± 109,890 units; *n* = 5 each; *p =* 0.218; Fig. 5e, f). On the other hand, FMRP labeling was significantly fainter in the IPL (CTR 746,590 ± 213,539 *vs.* VPA 667,232 ± 255,715 units; *n* = 5 each; paired *t* test, *p =* 0.050; Fig. 5e, f) and in the GCL of VPA mice (CTR 360,906 ± 80,118 units *vs.* VPA 265,780 ± 62,757 units; *n* = 5 each; paired *t* test, *p =* 0.020; Fig. 5e, f, all individual data in Additional file 1: Table S1). Regarding the retina as a whole, immunoblots showed significantly decreased FMRP content in the VPA mouse retina in comparison to CTR (CTR 0.70 ± 0.40 FMRP/beta-actin OD, *n* = 8 *vs.* VPA 0.31 ± 0.25 FMRP/beta-actin OD; *n* = 7; *p =* 0.05; Fig. 5i).

### VPA-exposed animals have altered retinal expression of proteins important for GABAergic function

**Fig. 6:**
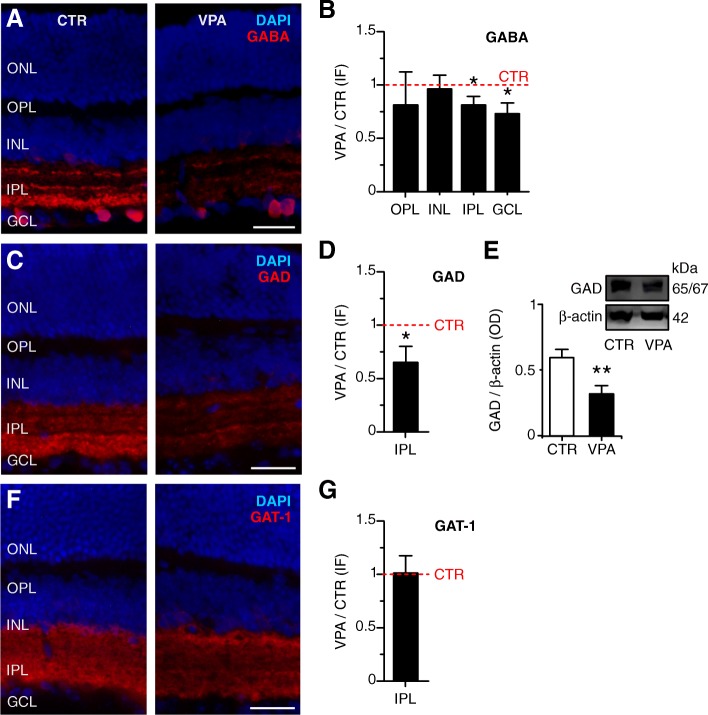
Decreased GABA and GAD expression in the VPA retina. **a** Photomicrographs from CTR and VPA retinal sections showing immunolabeling for GABA (red) and cell nuclei (DAPI, blue). The GABA antibody labels somas in the INL and GCL and bands of immunoreactive processes in the IPL. Scale bar = 25 μm. **b** Mean GABA fluorescence in VPA retinas relative to CTR (± SEM; *n =* 6 pairs) in the OPL (*p =* 0.735), INL (*p =* 0.425), IPL (*p = 0.030), and GCL (*p = 0.040). **c** Photomicrographs of sections from CTR and VPA retinas showing immunolabeling for GAD (red) and nuclei (DAPI, blue). GAD immunoreactivity is located in the IPL. Scale bar = 25 μm. **d** Mean GAD fluorescence in VPA retinas relative to CTR (± SEM; *n =* 5 pairs, *p = 0.037). **e** Left: Mean optical density (± SEM) of the GAD immunofluorescence in blots of CTR (white bar, *n =* 8) and VPA (black bar, *n =* 7) retinas, normalized to the beta-actin signal (**p = 0.007). Right: Representative GAD bands (65/67 kDa) and the corresponding beta-actin bands (42 kDa) of CTR *vs.* VPA. **f** Photomicrographs of sections from CTR and VPA retinas showing immunolabeling for GAT-1 (red) and nuclei (DAPI, blue). GAT-1 immunoreactivity is diffusely distributed throughout the IPL. Scale bar = 25 μm. **g** Mean GAT-1 signal in VPA retinas relative to CTR (± SEM; *n =* 5 pairs, *p =* 0.973). The red dashed lines in **b**, **d**, and **g** represent the value for CTR (= 1)

It was reported that GABA releasing neurons in the mouse retina are likely to be restricted to several subtypes of AC in both the INL and GCL and a subtype of interplexiform cell with processes in the OPL [38]. GABA immunoreactivity was very faint and did not differ between groups in the OPL (CTR 100,986 ± 46,894 fluorescence units *vs.* VPA 89,227 ± 93,380 units; *n* = 6 each; paired *t* test, *p =* 0.735) and in the INL (CTR 182,965 ± 57,403 fluorescence units *vs.* VPA 167,146 ± 51,914 units; *n* = 6 each; *p =* 0.425; Fig. 6a). However, VPA retinas presented decreased labeling in both IPL (CTR 671,074 ± 199,068 fluorescence units *vs.* VPA 545,808 ± 199,725 units; *n* = 6 each; paired *t* test, *p =* 0.030) and GCL (CTR 449,120 ± 106,054 fluorescence units *vs.* VPA 319,857 ± 110,109 units; *n* = 6 each; paired *t* test, *p =* 0.040; Fig. 6a, individual data in Additional file 1: Table S1). Figure 6b shows that the mean GABA-immunoreactive signal of VPA retinas after normalization was 0.81 ± 0.19 times that of CTR retinas in the IPL and 0.73 ± 0.23 times that of CTR in the GCL.

A decreased GABA content in cells and synapses might be caused by either diminished synthesis or increased release. In order to investigate the role of these two factors in our results, we studied the retinal expression of GAD (an enzyme involved in the synthesis of GABA) and of the neuronal GABA transporter (GAT-1), responsible for both uptake and transporter-mediated GABA release in the retina [39]. GAD immunoreactivity in CTR retinas was restricted to a diffuse labeling pattern in the IPL that was decreased in VPA mice (CTR 576,988 ± 230,994 fluorescence units *vs.* VPA 434,265 ± 313,477 units; *n* = 5 each; paired *t* test, *p =* 0.040; Fig. 6c, d); the IPL was a region in which GABA content was also reduced in VPA animals (Fig. 6a, b). Figure 6d shows that the mean GAD immunofluorescence of VPA retinas after normalization was 0.65 ± 0.34 times that of CTR in the IPL. The total retinal GAD content was also decreased in VPA mice (CTR 0.67 ± 0.20 GAD/beta-actin OD, *n* = 8 *vs.* VPA 0.36 ± 0.18 GAD/beta-actin OD, *n* = 7; *p =* 0.007; Fig. 6e). On the other hand, neither the GAT-1 immunoreactivity pattern nor its intensity was altered in VPA mice in relation to CTR; labeling was diffusely distributed throughout the IPL in both groups (CTR 749,768 ± 51,331 fluorescence units *vs.* VPA 745,521 ± 238,720 units; *n* = 5 each; paired *t* test, *p =* 0.973; Fig. 6f, g, individual data in Additional file 1: Table S1). Therefore, the GABA decrease found in VPA-exposed mice is likely to be due to its diminished synthesis rather than its increased release or reduced uptake**.**

## Discussion

In this paper, we show that VPA-exposed pups are smaller and lighter than CTR animals and present behavioral traits consistent with an ASD model. As previously shown by other groups [24, 25], intra-uterine exposure to VPA induces more anxiety-like behaviors and reduced social interest in male rodents, allowing us to consider them a valid phenotypic ASD model. Several structures and organs have been studied in these animals, such as the hippocampus [40], the cortex [41], and the liver [42], but this is the first study to explore the retina.

We here report that the ERGs of VPA mice are significantly altered in comparison to those of CTR animals. The a-wave amplitudes are smaller at higher light levels. This effect is unlikely to result from photoreceptor loss, since the thickness of the outer nuclear layer (ONL) is similar in both mouse groups (i.e., compare CTR and VPA photomicrographs in Figs. 5 and 6) and since the sensitivity (*k*) and slope (*n*) of the a-wave intensity-response relation are unchanged in the VPA model. Such a decrease in the maximal amplitude with no concomitant change in the sensitivity or gain of the ERG a-wave was also described in the *Fmr1*^−/−^ mouse, an animal model with ASD-like behaviors that lacks the FMRP protein, among other retinal alterations [43, 44], and points to the possibility that the phototransduction machinery within photoreceptors may be changed in the VPA model. Furthermore, since (i) the a-wave is contributed to by both rods and cones [30, 34], (ii) rods contribute more to the ERG at low light levels, whereas cones contribute more at higher light levels [30, 34], and (iii) our results show a larger effect at high light intensities, it is likely that cones are more affected than rods in VPA-exposed animals. This interpretation is supported by the finding in individuals with ASD that the light-adapted ERGs, which reflect cone and cone-driven activity in the retina, are more altered than dark-adapted ERGs, which reflect rod and rod-driven activity [45]. Further analogies with work in humans are however harder at this point, due to the scarcity of literature and to the small and inhomogeneous samples inherent to available studies.

On top of that, both the b-wave and the OPs appear normal in the VPA model, despite the smaller a-wave and possible decreased photoreceptor input to BCs and from BCs to ACs. These results indicate that adaptive changes are probably taking place in both synaptic layers of the retina in order to adjust the gain of these synapses and to compensate for the smaller photoreceptor input. Comparatively similar compensatory mechanisms in the signal transmission from the outer to the inner retina were also described in the ERGs of the *Fmr1*^−/−^ mouse [43, 44] and were shown to have very early onset in development [44]. Our ERG results are therefore consistent with the work of others and also with the altered expression of synaptic proteins in the outer and inner retina of VPA mice that we report here. However, one cannot at this point establish causality, because the exact function of several of these proteins is still unknown in the retina, as discussed below. It is of note, however, that the magnitude of alterations observed seems larger at the protein level than at the functional level, which raises the question of how substantial a structural change must be for it to translate into a sizeable effect.

Since ASD has been considered a synaptic disorder, the first molecule we analyzed was synapsin-1 (SYN-1), a member of the synapsin family of phosphoproteins essential for the fine-tuning of synaptic plasticity. SYN-1 is a crucial regulator of neurotransmitter release at pre-synaptic compartments of both excitatory and inhibitory conventional synapses (reviewed in [46]). An important role for molecular processes shaping higher brain functions was suggested by the observed phenotypes of synapsin *null* mutants. The absence of *syn* genes induced behavioral alterations in mice similar to those observed in ASD [47].

According to previous reports, SYN-1 is expressed by ACs the IPL, being absent from ribbon synapses in the outer and inner retina [36, 48]. It is important for the proper development of connections between cells in the INL and GCL [49]. The decreased SYN-1 expression that we find in VPA mice might indicate a less developed inner retina, with immature or decreased number of connections. Therefore, our experimental model produces in the retina a pattern of reduced SYN-1 expression reported in the pathogenesis of ASD [50].

It is known that mGluR5-enhanced activity mediates some of the behavioral characteristics observed in ASD patients, and mGluR5 antagonists have been studied as possible therapeutic agents for autism disorders [37]. Indeed, increased mGluR5 has been shown in the retinas of the *Fmr1*^*−/−*^ mouse [43], suggesting that it is likely to be a common feature of ASD models. We have also found increased mGluR5 immunoreactivity in the retinas of VPA-exposed mice. Retinal functions for mGluR5 are probably manifold. In the outer retina, pharmacological activation of group I mGluRs (which include mGluR1 and mGluR5) leads to closure of the K^+^ conductance in Müller cells [51]; because glutamate uptake from these glial cells is controlled both by voltage and by their internal K^+^ concentration [52, 53], it was suggested that increased mGluR5 expression could lead to changes in local glutamate homeostasis [54]. In the inner retina, group I mGluRs may modulate GABA release from ACs [39]; mGluR5, specifically, appears to have effects onto GABA_A_ receptor function in cultured ACs [55]. Therefore, the increased expression of mGluR5 in VPA animals could potentially induce changes in both glutamatergic and GABAergic signaling pathways.

Several proteins related to the GABAergic system are reduced in individuals with fragile X syndrome [56, 57]. In our study, we observed a decrease in GABA and GAD expression in the IPL of animals prenatally exposed to VPA, but not in neuronal transporter GAT-1, suggesting that the GABAergic activity in the retina could be decreased due to diminished GABA synthesis. Alternatively, the higher expression of mGluR5 in VPA retinas could lead to an increase in transporter-mediated GABA release from ACs [39], which would in turn translate into GABA content inside these neurons.

Genetic mutations that lead to decreased FMRP cause fragile X syndrome, the most commonly known inherited form of intellectual disability (ID), which has an ASD diagnosis associated in some patients [58]. FMRP is an RNA-binding protein that regulates local protein synthesis in the synapse when dephosphorylated by mGluR5 [59]. Low FMRP expression causes the excessive synthesis of proteins that should only be translated upon mGluR5 activation, a phenomenon that leads to protein accumulation and consequent synaptic disturbance [59, 60]. FMRP is present in the retina and its expression is lower in dark-adapted conditions than after light adaptation [61]. Therefore, FMRP expression changes might lead to alterations in physiological responses of retinal neurons. In fact, *Fmr1*^−/−^ animals present functional changes in both outer and inner retinal anatomy and function [43, 44]. Accordingly, our results show that not only does the retina of the VPA-exposed mice express less FMRP than control mice, but it also displays morphological and physiological features similar to the *Fmr1*^−/−^ model.

## Conclusion

In summary, we conclude that male adolescent mice born from VPA-injected dams display ASD-like behaviors and show altered expression of synaptic, glutamatergic, and GABAergic markers in both synaptic layers of the retina. Importantly, these animals present significant alterations in the full-field scotopic ERG, suggesting photoreceptor dysfunctions, with cones probably more affected than rods. These functional alterations are accompanied by compensatory mechanisms in the post-receptoral circuitry, which are consistent with the structural changes that we report in this model and in the literature dealing with other ASD models [43, 44]. It remains to be shown how these animals respond to different stimulation protocols, such as the photopic and pattern ERGs, which test cone-driven pathways and the inner retina, respectively [62, 63]. Also, since the actual role of the proteins related to ASD in the retina is still unknown, it would be interesting to examine the retinal anatomy and physiology of transgenic mice lacking or overexpressing these proteins in order to establish their importance for retinal function and vision. Finally, our results clearly confirm that the retina is an accessible window to study brain wiring and function in both health and disease. It can thus provide invaluable insight into the underpinnings of psychiatric disorders and subserve translational approaches aiming to diagnose and treat such conditions.

## Additional file

Additional file 1: **Figure S1.** Experimental design. IHC: immunohistochemistry; WB: Western blotting; BH: behavioral experiments; ERG: electroretinogram. **Figure S2.** Chambers used for the behavioral assays in our study. **Figure S3.** VPA-exposed animals are smaller and lighter than CTR mice. **Video S1.** Representative CTR animal in the open field test. The video shows the first 3 min of exploration. **Video S2.** Representative VPA animal in the open field test. The video shows the first 3 min of exploration. **Video S3.** Representative CTR animal in the social interaction test. **Video S4.** Representative VPA animal in the social interaction test. **Table S1.** Arbitrary fluorescence units obtained from individual retinas. Results from CTR and VPA pairs (shown in the same line) were processed together on the same day. Statistics were performed through a paired Student’s t test to take into consideration the fact that results in different lines are attributed to signal variation between experiments. (DOCX 6386 kb)

## Abbreviations

AC: Amacrine cell
ASD: Autism spectrum disorders
CNS: Central nervous system
ERG: Electroretinogram
FMRP: Fragile X mental retardation protein
GABA: Gamma-aminobutyric acid
GAD: Glutamic acid decarboxylase
GAT-1: Sodium- and chloride-dependent GABA transporter 1
GCL: Ganglion cell layer
GSK-3 beta: Glycogen synthase kinase-3 beta
HDAC: Histone deacetylase
INL: Inner nuclear layer
IPL: Inner plexiform layer
mGluR5: Metabotropic glutamate receptor 5
NMDA: *N*-methyl-D-aspartate receptor
OD: Optical density
ON BC: Depolarizing bipolar cell
ONL: Outer nuclear layer
OPL: Outer plexiform layer
OPs: Oscillatory potentials
P30: Postnatal day 30
SYN-1: Synapsin-1
VPA: Valproic acid
