# Supplementary material for: Retinal alterations in a pre-clinical model of an autism spectrum disorder
Source: Mol Autism. 2019 Apr 15;10:19. doi: 10.1186/s13229-019-0270-8 (PMC6466731; doi:10.1186/s13229-019-0270-8)
Supplement: Supplementary file 1 — Figure S1. Experimental design. IHC: immunohistochemistry; WB: Western blotting; BH: behavioral experiments; ERG: electroretinogram. Figure S2. Chambers used for the behavioral assays in our study. Figure S3. VPA-exposed animals are smaller and lighter than CTR mice. Video S1. Representative CTR animal in the open field test. The video shows the first 3 min of exploration. Video S2. Representative VPA animal in the open field test. The video shows the first 3 min of exploration. Video S3. Representative CTR animal in the social interaction test. Video S4. Representative VPA animal in the social interaction test. Table S1. Arbitrary fluorescence units obtained from individual retinas. Results from CTR and VPA pairs (shown in the same line) were processed together on the same day. Statistics were performed through a paired Student’s t test to take into consideration the fact that results in different lines are attributed to signal variation between experiments. (DOCX 6386 kb) [file 13229_2019_270_MOESM1_ESM.docx]

**Additional Materials**

Figure S1 depicts a scheme with the numbers of injected dams, dams with litters, animals in each litter, and their respective experimental group assignments. The rightmost squares depict the final numbers of mice in each group and type of experiment. In all cases, *n*s are within parentheses. Notice that *n*s correspond to the total number of animals initially obtained. Some samples/animals were lost during the experimental procedures; final *n*s are given in the text.

**
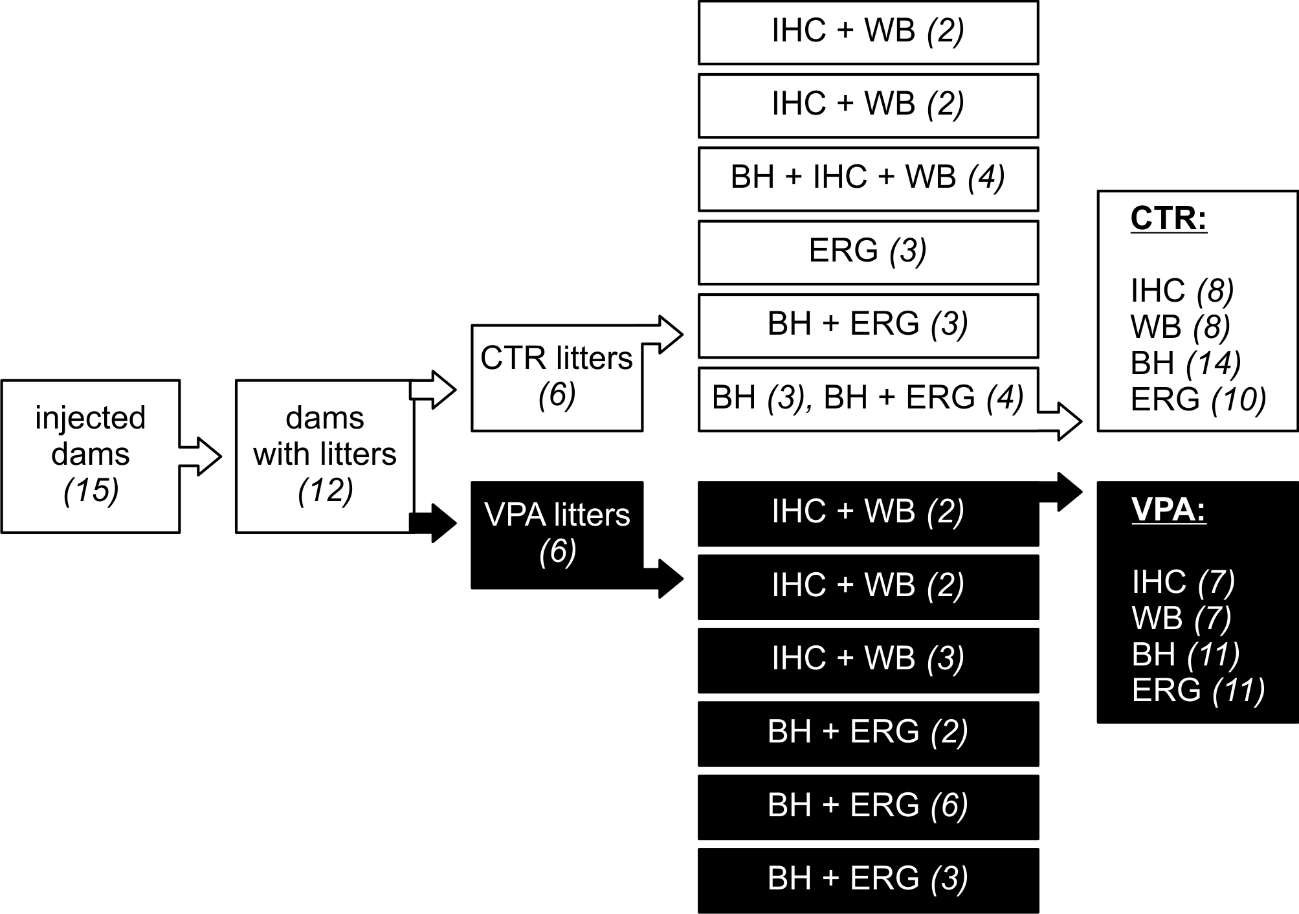
**

**Figure S1 –** **Experimental design.** IHC: immunohistochemistry; WB: Western blotting; BH: behavioral experiments; ERG: electroretinogram.

The left panel of Figure S2 shows the open field. Peripheral (quadrants near the walls) and central area are indicated by the blue and red squares, respectively. The right panel of Figure S2 shows the social interaction arena. The interaction and total areas are indicated by the red and green squares, respectively.


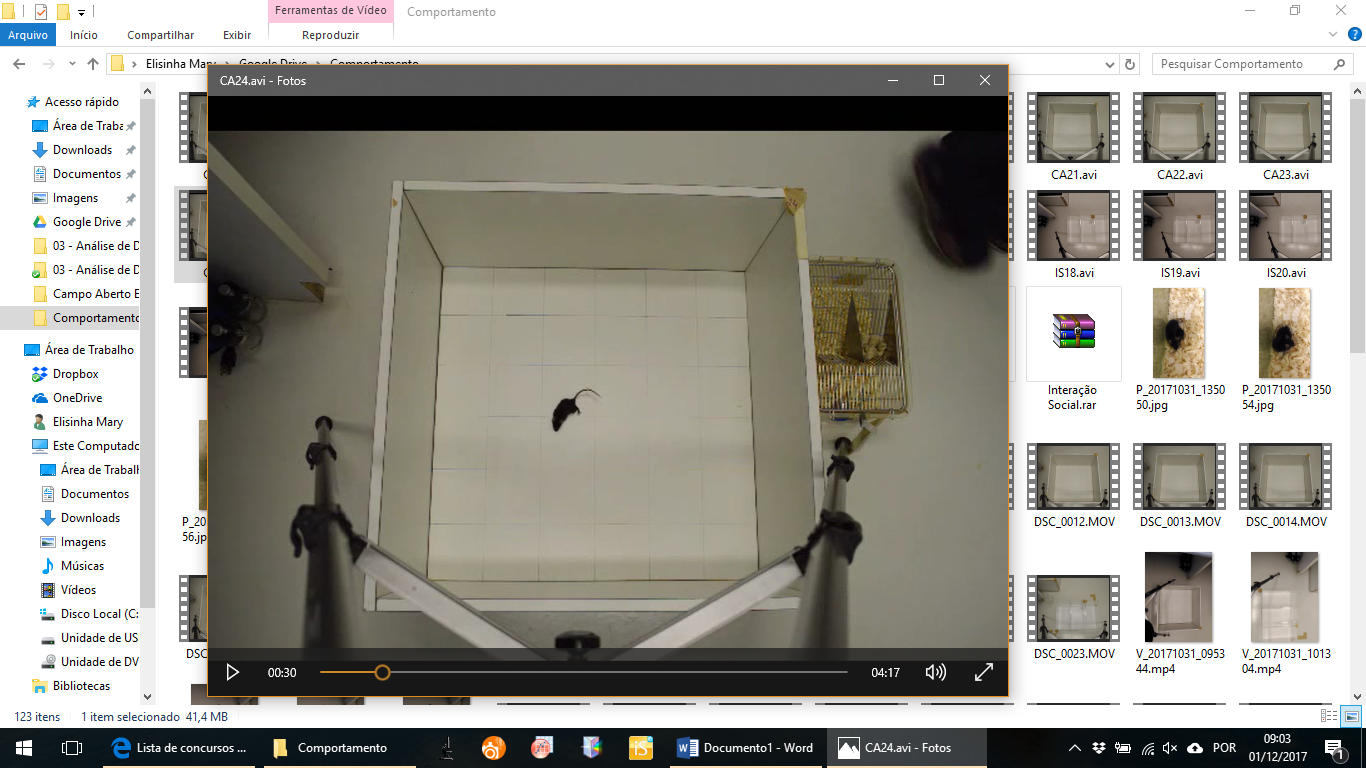

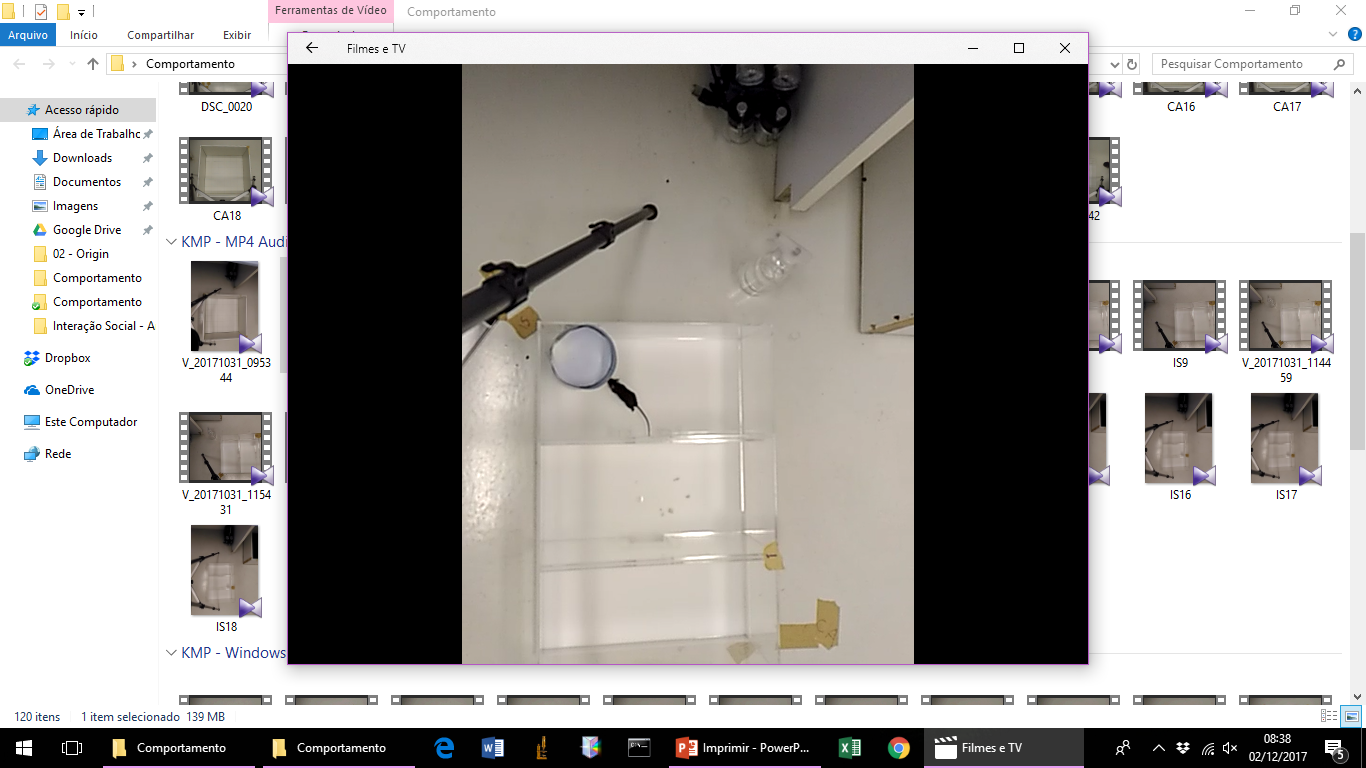


**Figure S2** – **Chambers used for the behavioral assays in our study.**

Panel (A) of Figure S3 is a photograph of a VPA litter showing animals of different body sizes, with hair loss and crooked tail (red arrow). The left side of panel (B) shows the mean weights of the CTR and VPA mice that underwent the ERG (CTR: 19.1 ± 3.1 g, *n* = 10; VPA: 14.0 ± 4.6 g, *n* = 11; ***p* = 0.008). The right side of panel (B) presents the mean electrode impedances (left and right corneal electrodes for each mouse, values for three animals are missing) for CTR and VPA mice that underwent the ERG (CTR: 7.5 ± 1.4 kΩ, *n* = 16; VPA: 7.9 ± 2.2 kΩ, *n* = 20; *p* = 0.501). Error bars are SEM for both panels. Although weights differed between groups, impedances did not, indicating that the effects we found in the ERG were not due to impedance differences (i.e. eye size difference) between groups. Panel (C) is a photograph of a VPA pup with small size, low weight, and two sites of crooked tail (red arrows). Panel (D) is a photograph of a CTR pup and a VPA pup of the same age, for comparison. Red arrow indicates crooked tail.


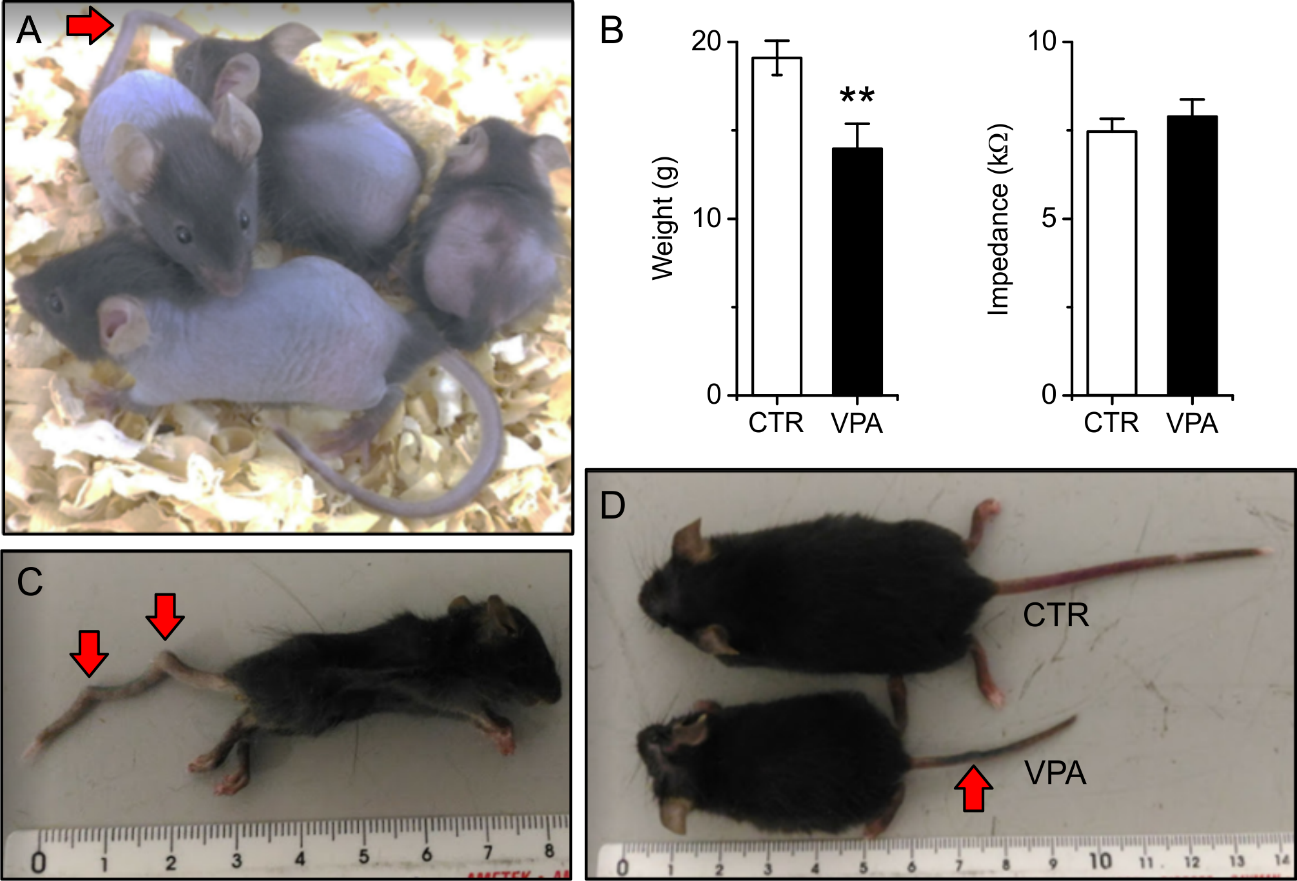


**Figure S3 – VPA-exposed animals are smaller and lighter than CTR mice.**

**Video S1 Still and Link:**


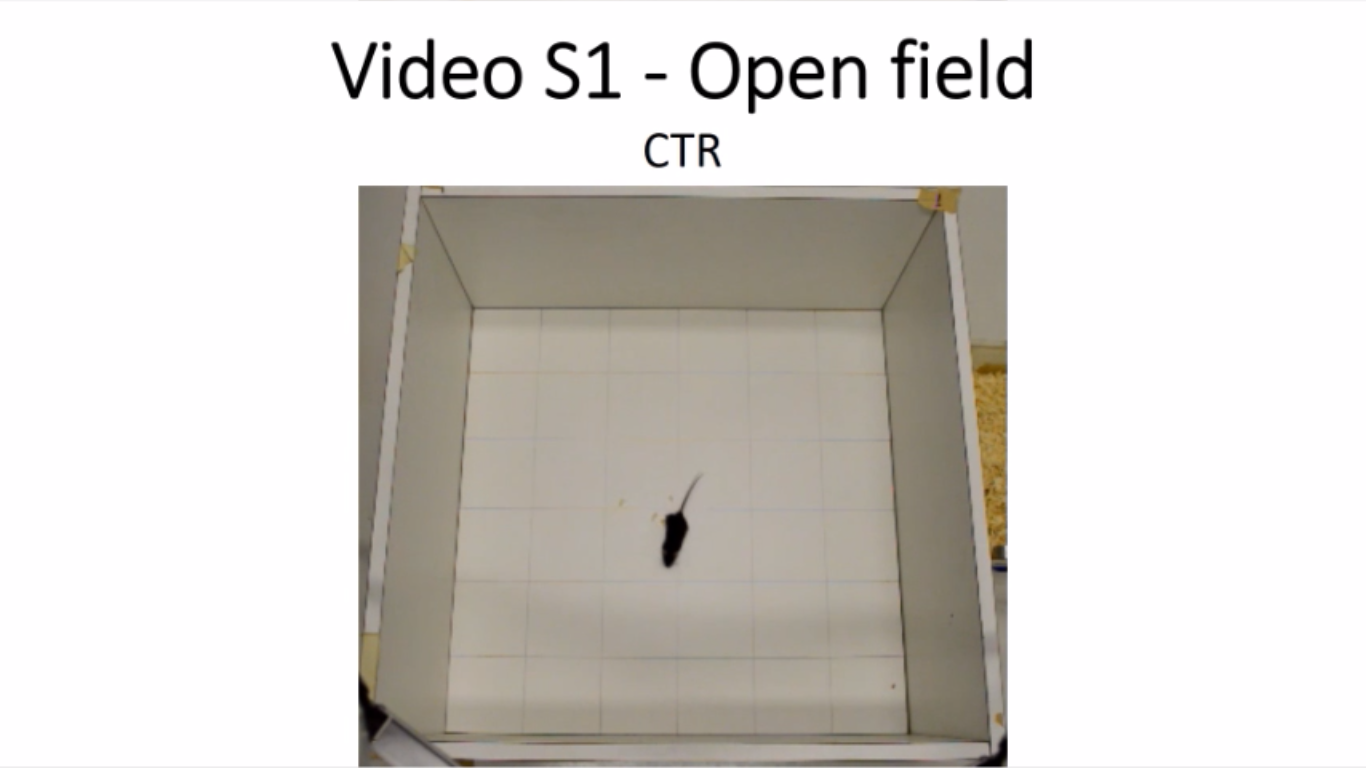


**Video S1 – Representative CTR animal in the open field test.** The video shows the first 3 min of exploration. <https://youtu.be/caGSLOZ8VMQ>

**Video S2 Still and Link:**


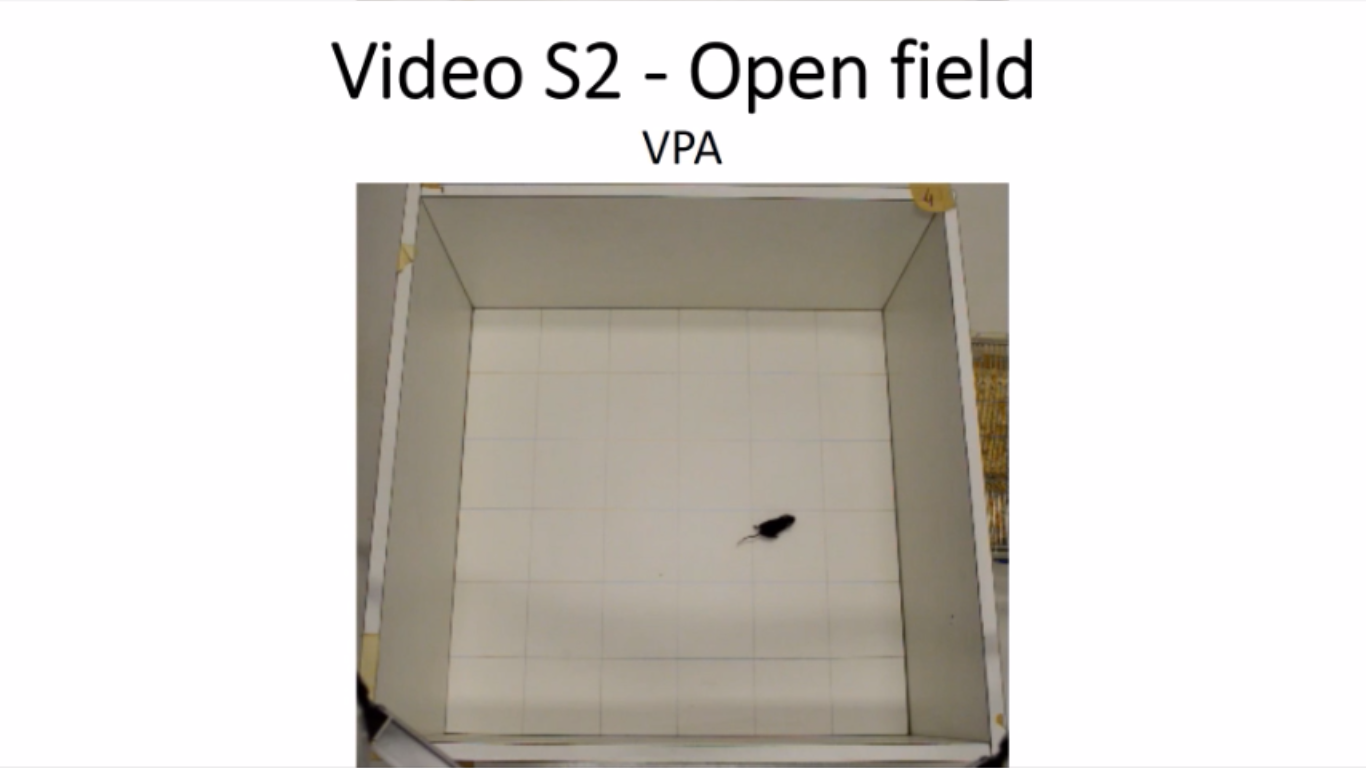


**Video S2 – Representative VPA animal in the open field test.** The video shows the first 3 min of exploration. <https://youtu.be/TRrjPU-UOK4>

**Video S3 Still and Link:**


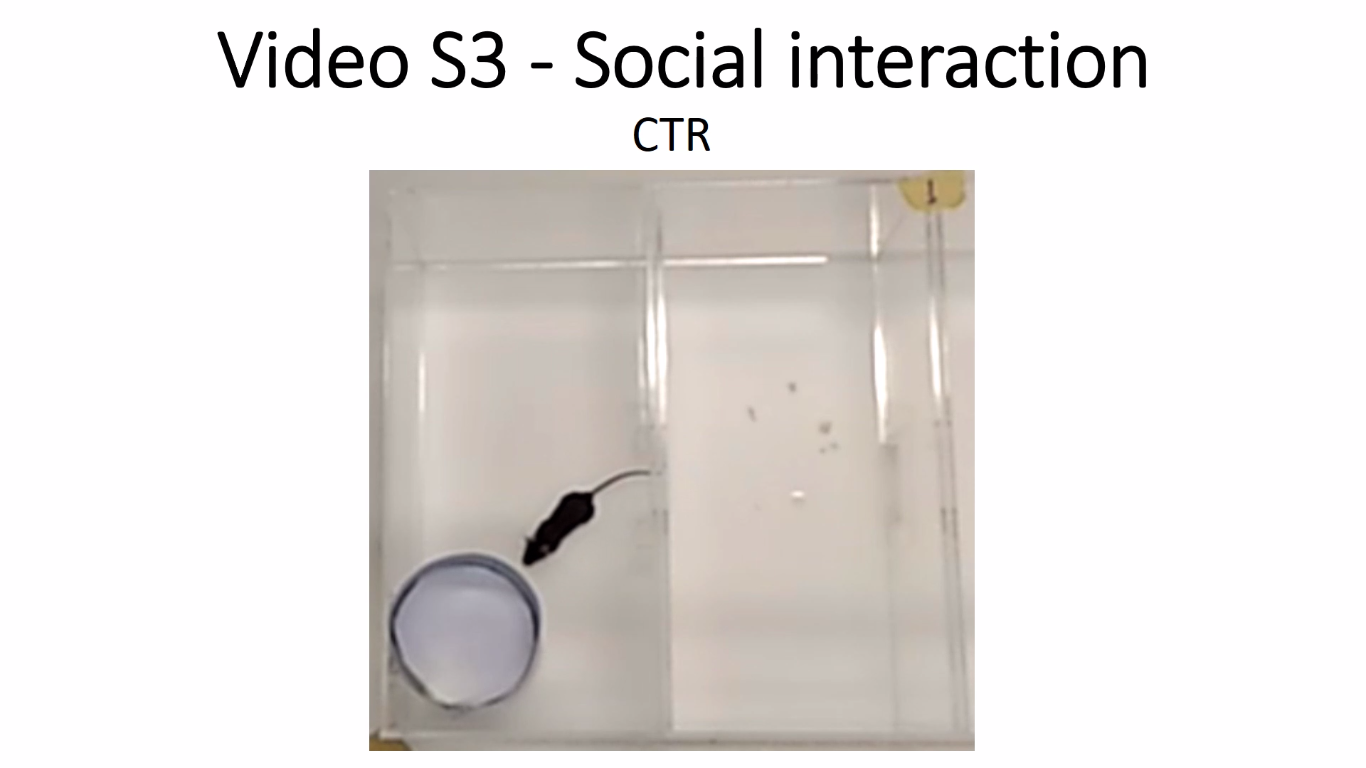


**Video S3 – Representative CTR animal in the social interaction test.** <https://youtu.be/LLDEN5rDDxw>

**Video S4 Still and Link:**


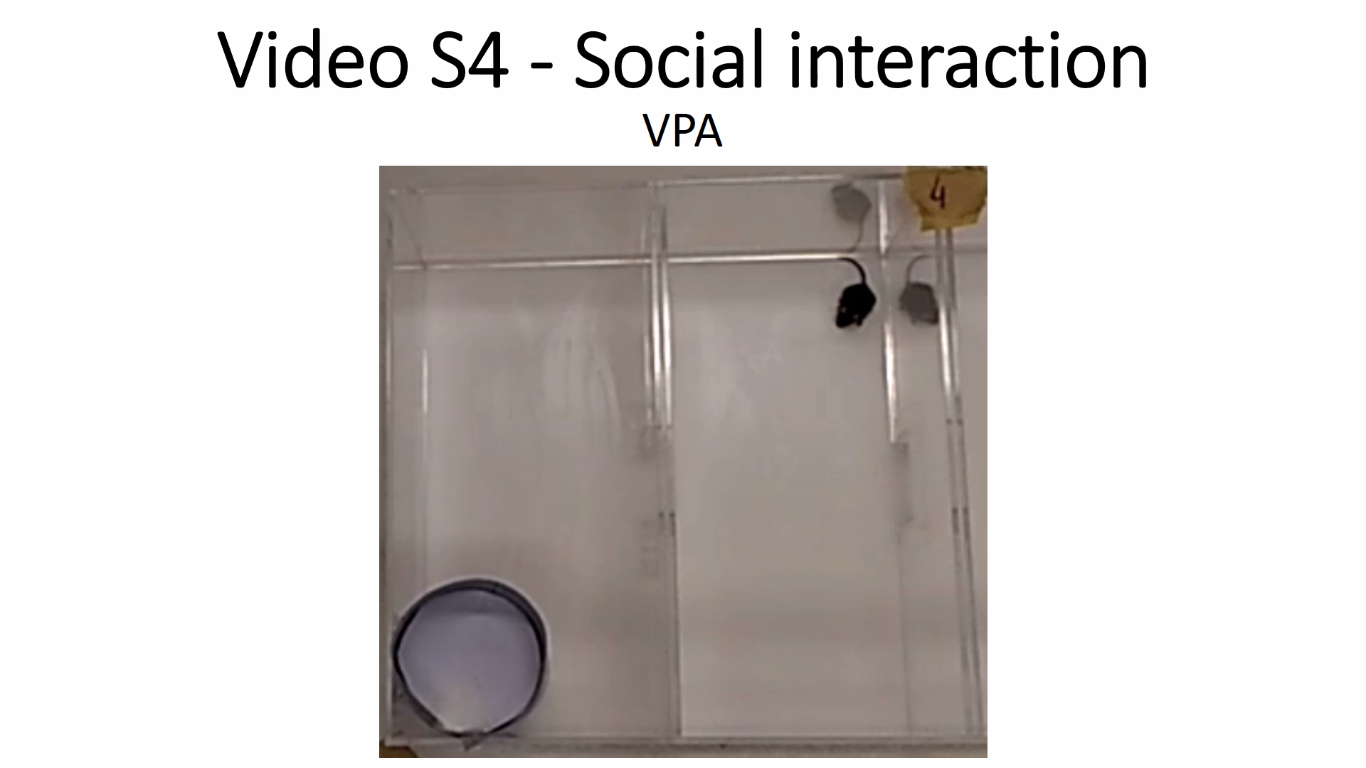


**Video S4 – Representative VPA animal in the social interaction test.** <https://youtu.be/WzbavRE5Zo0>

| **SYN-1 – IPL** | |  |  |  |  |  |  |
| --- | --- | --- | --- | --- | --- | --- | --- |
| **CTR** | **VPA** |  |  |  |  |  |  |
| 265822 | 126818 |  |  |  |  |  |  |
| 279923 | 111274 |  |  |  |  |  |  |
| 901921 | 828080 |  |  |  |  |  |  |
| 814925 | 441219 |  |  |  |  |  |  |
| 625650 | 457394 |  |  |  |  |  |  |
| **mGluR5 – OPL** | | **mGluR5 – IPL** | |  |  |  |  |
| **CTR** | **VPA** | **CTR** | **VPA** |  |  |  |  |
| 237080 | 383751 | 333265 | 450349 |  |  |  |  |
| 240598 | 439739 | 289880 | 317000 |  |  |  |  |
| 445202 | 859485 | 360292 | 513593 |  |  |  |  |
| 236606 | 442471 | 338774 | 540021 |  |  |  |  |
| 175837 | 209392 | 221197 | 314579 |  |  |  |  |
| 401575 | 614115 | 600656 | 950455 |  |  |  |  |
| **FMRP – OPL** | | **FMRP - INL** | | **FMRP - IPL** | | **FMRP - GCL** | |
| **CTR** | **VPA** | **CTR** | **VPA** | **CTR** | **VPA** | **CTR** | **VPA** |
| 193136 | 184395 | 106532 | 98465 | 1046024 | 997807 | 429276 | 235232 |
| 175012 | 263672 | 112996 | 106607 | 510357 | 341887 | 437266 | 354776 |
| 129662 | 247911 | 67810 | 200888 | 773810 | 656305 | 239598 | 184351 |
| 186679 | 428089 | 166709 | 371020 | 828069 | 822226 | 339691 | 275624 |
| 230064 | 239102 | 209416 | 208203 | 574689 | 517935 | 358699 | 278915 |
| **GABA – OPL** | | **GABA - INL** | | **GABA - IPL** | | **GABA - GCL** | |
| **CTR** | **VPA** | **CTR** | **VPA** | **CTR** | **VPA** | **CTR** | **VPA** |
| 155796 | 138922 | 183939 | 116765 | 683240 | 454900 | 458779 | 466909 |
| 111063 | 250511 | 91591 | 134243 | 429649 | 256704 | 329878 | 189139 |
| 109927 | 56254 | 162701 | 192500 | 612589 | 486629 | 487085 | 211350 |
| 113939 | 77970 | 268907 | 259908 | 966257 | 849371 | 538553 | 418470 |
| 101618 | 5749 | 198705 | 150088 | 824077 | 650734 | 567347 | 330592 |
| 13570 | 5954 | 191947 | 149370 | 510629 | 576509 | 313076 | 302684 |
| **GAD – IPL** | |  |  |  |  |  |  |
| **CTR** | **VPA** |  |  |  |  |  |  |
| 345328 | 108291 |  |  |  |  |  |  |
| 316564 | 88006 |  |  |  |  |  |  |
| 803921 | 754617 |  |  |  |  |  |  |
| 761016 | 575705 |  |  |  |  |  |  |
| 658110 | 644707 |  |  |  |  |  |  |
| **GAT-1 – IPL** | |  |  |  |  |  |  |
| **CTR** | **VPA** |  |  |  |  |  |  |
| 740221 | 411762 |  |  |  |  |  |  |
| 802679 | 602118 |  |  |  |  |  |  |
| 794147 | 902048 |  |  |  |  |  |  |
| 736146 | 805628 |  |  |  |  |  |  |
| 675648 | 1006051 |  |  |  |  |  |  |

**Table S1 –** **Arbitrary fluorescence units obtained from individual retinas.** Results from CTR and VPA pairs (shown in the same line) were processed together on the same day. Statistics were performed through a paired Student’s *t* test to take into consideration the fact that results in different lines are attributed to signal variation between experiments.
